# Moving LLM evaluation forward: lessons from human judgment research

**DOI:** 10.3389/frai.2025.1592399

**Published:** 2025-05-27

**Authors:** Andrea Polonioli

**Affiliations:** Coveo, Quebec City, QC, Canada

**Keywords:** LLM, generative AI (GenAI), hallucinations, AI in business, human judgment, judgment and decision making, heuristics & biases

## Abstract

This paper outlines a path toward more reliable and effective evaluation of Large Language Models (LLMs). It argues that insights from the study of human judgment and decision-making can illuminate current challenges in LLM assessment and help close critical gaps in how models are evaluated. By drawing parallels between human reasoning and model behavior, the paper advocates moving beyond narrow metrics toward more nuanced, ecologically valid frameworks.

## 1 Introduction

Large Language Models (LLMs) have become central to the progress of artificial intelligence, powering advances across industries—from healthcare and education to legal analysis and creative writing (Chowdhery et al., 2022; Touvron et al., 2023). The public release of ChatGPT in 2022 marked a turning point, introducing LLMs into everyday discourse and positioning them as general-purpose intelligence systems. Yet despite their impressive versatility, these models often produce surprising errors, raising persistent questions about how to evaluate their reliability and adaptability (Bishop, 2021).

A growing ecosystem of benchmarks has emerged to address this challenge. Factuality assessments such as FELM (Chen et al., 2023), code-focused tasks like HumanEval (Chen et al., 2021), and domain-specific evaluations like SWE-bench Verified (Jimenez et al., 2023) each offer partial insight into model capabilities. Ranking-based platforms like Chatbot Arena (Zheng et al., 2023) have further shaped public perception, rewarding models that perform well in direct comparison. Yet these evaluation strategies remain fragmented and narrow, often incentivizing superficial improvements rather than generalizable progress.

Promising developments within the deep learning community have begun to address these limitations. Notably, Martin et al. (2021) present a framework for evaluating neural networks using structural metrics derived from the models’ own weight matrices. Building on theoretical results by Martin and Mahoney (2021), their approach offers a means of assessment that does not rely on external test data. It introduces a different kind of benchmark—one that focuses on the internal properties of a model and the distribution of capacity across its architecture. In doing so, it offers a more nuanced perspective on model quality, extending beyond the fragmented and task-bound metrics that dominate much of today’s evaluation landscape. Nevertheless, such contributions have yet to significantly shape the broader discourse, which remains largely driven by surface-level performance and high-profile failure cases (e.g., Silberling, 2024).

Consider this seemingly simple exchange:

Human: “How many R’s are in the word *strawberry*?”

LLM: “There are two.”

Human: “Actually, there are three—one in the middle and two at the end.”

LLM: “No, count again.”

LLMs frequently fail at these kinds of counting tasks, producing confident but incorrect responses. Such errors raise concerns not only about model precision but also about the deeper mechanics of how these systems handle symbolic information and logical sequence processing. Do these failures reflect minor blind spots in token processing, or do they expose more fundamental architectural limitations? Could such mistakes result from asking the wrong kind of question—or using the wrong kind of evaluation? Are these isolated quirks, or signs of broader, generalizable weaknesses? And do different models exhibit systematically different error patterns? Recent evidence suggests yes – different models can have distinctive failure profiles. For example, Martin et al.’s analysis (2020) indicates that model architecture and training influence the types of errors a network is prone to.

Once these questions are raised, it becomes clear that they echo long-standing debates in the study of human judgment. For decades, cognitive scientists have explored how people process information, why they make systematic errors, and whether such errors signal irrationality or adaptive trade-offs. Concepts like bounded rationality (Simon, 1955) and ecological validity (Gigerenzer and Todd, 1999) helped reframe these debates—moving beyond binary success/failure judgments toward more nuanced, context-sensitive models of reasoning. These same ideas, we argue, can enrich the way we approach LLM evaluation.

This paper contends that advancing LLM evaluation requires drawing from the intellectual history of human judgment research. By moving beyond narrow benchmarks and reductive metaphors toward frameworks that foreground trade-offs, context, and structured interventions, we can build a more robust and empirically grounded understanding of what these models can - and cannot - do.

## 2 Accuracy does not speak with one voice

Just as research in human judgment and decision-making has long been shaped by influential metaphors (e.g., “cognitive illusions” and “biases”), the evaluation of LLMs has similarly gravitated toward evocative language. In particular, “hallucination” has emerged as a dominant descriptor of model error. While some scholars have proposed alternatives like “confabulation,” drawn from neuropsychology to describe plausible but incorrect responses in the absence of sufficient information (Smith et al., 2023), others—such as Brender (2023)—have rejected anthropomorphic metaphors altogether, warning that terms like *hallucination* risk projecting human cognitive assumptions onto fundamentally different systems.^1^

The issue with such metaphors is not only that they introduce conceptual baggage or polarize discussion; more critically, they oversimplify the multifaceted nature of model failure. LLM outputs do not merely succeed or fail in binary terms—accuracy manifests across different dimensions. Some errors reflect misalignment with external truth (factuality), while others arise from internal inconsistency, poor calibration, or sensitivity to prompt phrasing.

Hammond’s (2007) distinction between coherence and correspondence in human judgment offers a useful lens. Coherence refers to internal consistency—how well a model’s outputs logically hang together. This concept is central to the heuristics-and-biases tradition, which often highlights deviations from logical norms (e.g., the conjunction fallacy; Kahneman and Tversky, 1983). Correspondence, by contrast, focuses on alignment with external reality and predictive success, as seen in ecological approaches like fast-and-frugal heuristics (Gigerenzer and Todd, 1999; Polonioli, 2014, 2016). For example, the recognition heuristic can help people make accurate predictions in uncertain environments despite limited information.

Crucially, coherence and correspondence do not always align (Arkes et al., 2016; Katsikopoulos, 2009). Coherence-based evaluations often cast human reasoning in a negative light, while correspondence-based approaches highlight when heuristics yield adaptive, real-world performance. This tension has been instrumental in reshaping how we assess rationality, and it offers a valuable precedent for LLM evaluation. Polonioli (2015) further argues that the coherence–correspondence distinction, while useful, does not exhaust the complexity of cognitive evaluation. Other dimensions—such as context sensitivity and calibration—also matter. As Nisbett and Wilson (1977) famously showed, human judgments are heavily influenced by contextual cues. LLMs exhibit similar fragility: minor prompt variations can yield dramatically different outputs, yet few benchmarks test this.

Despite a growing ecosystem of benchmarks, most focus overwhelmingly on correspondence. Datasets such as FELM (Factuality Evaluation of Large Language Models; Chen et al., 2023) or TruthfulQA (Lin et al., 2022) measure accuracy relative to known facts. These tools are valuable—but they neglect coherence-related errors, such as when models contradict themselves or generate answers that do not align with their own justifications.

Several recent studies hint at the importance of coherence, though not always explicitly. For example, Wang et al. (2022), in Self-Consistency Improves Chain-of-Thought Reasoning in Language Models, show that averaging a model’s answers across multiple reasoning paths often improves correctness—suggesting that internal consistency may correlate with better performance. Zhou et al. (2022), in *Least-to-Most Prompting Enables Complex Reasoning*, point out that LLMs sometimes arrive at correct answers via logically invalid chains—indicating that output correctness does not always reflect processing quality.

Other work more directly engages with internal inconsistency. Madaan et al. (2023), in Self-Refine: Iterative Refinement with Self-Feedback, explore prompting models to revise their own outputs—a method that frequently surfaces contradictions and logical analysis failures. Meanwhile, Macmillan-Scott and Musolesi (2024), in Biases and Fallacies in Large Language Models: A Human Reasoning Perspective, test LLMs on known human reasoning biases. Their findings show that while models can replicate certain fallacies, they often do so inconsistently or incoherently—further demonstrating that LLM failure modes do not map cleanly into human patterns.

Despite these developments, there is still no large-scale benchmark dedicated to assessing coherence in LLMs. This is a critical gap. If coherence is key to evaluating the quality of *how* models arrive at answers, then the absence of such a benchmark skews our understanding of model behavior and limits opportunities for targeted improvement. A coherence benchmark would bring at least three benefits:

1. Clarify the coherence–correspondence relationship: It would help disentangle cases where models generate correct answers for the wrong reasons—or coherent but incorrect responses.
2. Test generalization more meaningfully: Stable, consistent reasoning is likely to be more robust across prompt variations and domains.
3. Enable structured interventions: Coherence metrics could guide improvements like chain-of-thought prompting, instruction tuning, or self-verification.

In the same way that coherence and correspondence may not capture the full spectrum of human judgment, evaluating the intrinsic properties of language models offers an important complement to these dimensions. For instance, Martin et al. (2021) propose assessing neural networks through heavy-tailed spectral properties of their weight matrices. These structural indicators have been shown to correlate strongly with generalization performance across models - even in the absence of traditional test data. By analyzing a model’s internal structure, such methods offer a perspective that treats the model itself as data, complementing coherence-based evaluation with a view from the inside. This line of work reinforces our broader argument: that advancing LLM evaluation requires diverse and scalable approaches- those that assess both behavior externally and structure internally.

In short, just as the study of human cognition matured by expanding its understanding of rationality, LLM evaluation must move beyond narrow factuality checks. Accuracy does not speak with one voice—and understanding how models perform is central to grasping their capabilities and limitations. A dedicated, scalable coherence benchmark would mark an important step forward, as would further emerging criteria that focus on a model’s internal characteristics.

## 3 Assessing LLMs through the lens of bounded rationality

Much like human cognition, LLMs operate under resource constraints. They must balance competing objectives—accuracy, latency, compute efficiency, and cost. This mirrors what Herbert Simon (1955) described as bounded rationality: the idea that decision-makers (including artificial systems) rarely have unlimited time or resources and therefore rely on heuristics to make “good enough” decisions under constraint, rather than always optimizing for perfect accuracy.

This framework offers a compelling analogy for how we should evaluate LLMs. While current evaluation metrics often emphasize static measures—such as factual correctness or performance on fixed tests—they typically ignore the computational trade-offs that define real-world deployment. For instance, high-performing models like GPT-4 Turbo or Anthropic’s Claude 3 (Opus) may deliver excellent benchmark results, but they require vast GPU memory, distributed inference infrastructure, and expensive hardware acceleration. These systems are optimized for capability, not efficiency.

Meanwhile, smaller or more efficient models (e.g., Mistral-7B, DeepSeek-V2, or Phi-2) can deliver near-state-of-the-art performance on select tasks with significantly lower resource usage. In latency-sensitive applications (such as customer support or real-time decision aids), a slightly less accurate but immediate response may be more valuable than a more accurate yet delayed one.

The recent development of DeepSeek R2 in 2025 exemplifies this trade-off. Developed to be cost-effective and deployable on relatively constrained hardware, the model prioritizes throughput and latency over marginal gains in benchmark accuracy (Baptista et al., 2025). Similarly, new inference strategies like vLLM and GGUF-based quantization (e.g., running LLaMA-2 13B at 4-bit precision) show a growing interest in efficient deployment rather than leaderboard dominance.

Yet most public evaluation frameworks overlook these constraints, focusing almost exclusively on benchmark-based correctness. As a result, they fail to capture the resource–accuracy trade-off that is central to many applied AI systems. Just as bounded rationality urges us to assess human decision-making in light of ecological constraints, LLM evaluation should recognize that a model’s real-world utility depends not only on what it gets right, but also on what it achieves within the limits of time, compute, and memory.

In short, the bounded rationality perspective invites us to ask different questions about LLMs: not only “How accurate is this model?” but also “How effective is it under pressure?” and “How well does it scale when resources are tight?” Incorporating such perspectives is crucial. Without it, LLM benchmarks risk promoting models that are academically impressive but operationally impractical.

## 4 Rethinking generality: lessons from ecological rationality

A longstanding critique from Gigerenzer and Todd (1999) is that many so-called cognitive “biases” identified by the heuristics-and-biases tradition arose from using abstract or ecologically invalid tasks. When tested in contexts that mirrored real-world decision-making—such as using natural frequencies instead of probabilities—many biases disappeared. This insight is highly relevant to today’s conversations around LLMs: Are we evaluating these systems with benchmarks and tasks that reflect their intended real-world use.

The implications extend beyond benchmarking. The dominant narrative in AI assumes that generality is the hallmark of intelligence, with AGI (artificial general intelligence) as its ultimate form. But findings from ecological rationality and evolutionary psychology offer a different view: intelligence is about efficiency and adaptiveness within specific environments—not universal competence. Human cognition relies on specialized heuristics tailored to particular tasks and constraints. Similarly, recent trends in LLM research point toward a resurgence of task-specific optimization over raw generalization.

Concrete examples from the LLM landscape support this. For instance:

- Med-PaLM (Singhal et al., 2022) – a model fine-tuned on medical Q&A – outperforms general-purpose models like GPT-3.5 on domain-specific benchmarks such as USMLE-style exam questions.
- BloombergGPT (Wu et al., 2023), trained on a blend of financial news, filings, and proprietary data, significantly improves performance on finance-related NLP tasks compared to general models.
- WizardCoder (Xu et al., 2023) – a specialized coding assistant – can outperform a general LLM like ChatGPT on code generation and bug-fixing tasks.
- OpenAI’s rumored *“Strawberry”* model (referred to unofficially by researchers) reportedly emphasizes logical consistency and chain-of-thought reasoning over general fluency, aiming to improve structured problem-solving.

Moreover, retrieval-augmented generation (RAG) architectures (Lewis et al., 2020) are increasingly used to bring domain-specific grounding into LLMs—especially in legal, medical, and enterprise contexts—underscoring the need for environment-aware adaptation.

These developments challenge the assumption that general-purpose models are universally superior. Instead, they highlight the importance of aligning model design, training, and evaluation with the ecological context in which models operate. Thus, just as ecological validity reshaped how we understand human reasoning, it should also reshape how we evaluate LLMs. Benchmarks must reflect context-specific demands, and success should be defined in terms of fit-for-purpose performance, not abstract generality. Without this shift, we risk misjudging the capabilities—and limitations—of these increasingly central AI systems.

## 5 Task redesign and structural interventions in LLM research

If the previous section raised concerns about representativeness and cross-task generalization, this one turns to robustness: Why do LLMs fail, and how can their outputs be systematically improved?

A central lesson from human judgment research is that performance can often be improved not by altering individual cognition directly, but by modifying the structure of the task or environment. This insight underpins the distinction between nudging and boosting—two families of interventions aimed at facilitating better decisions. Boosting, in particular, emphasizes durable, transparent improvements via structural changes to how information is presented (Hertwig and Grüne-Yanoff, 2017). A classic illustration comes from Gigerenzer and Hoffrage (1995), who showed that presenting statistical information as natural frequencies (e.g., “8 out of 10”) rather than probabilities dramatically enhances diagnostic analysis. Such insights have informed practice in domains as varied as medicine, law, and public policy (Gigerenzer et al., 2007).

A similar structuralist perspective is emerging in LLM research. Interventions like prompt engineering, instruction tuning, and retrieval-augmented generation (RAG) have been shown to significantly improve model outputs without modifying the underlying weights. For example, Wei et al. (2022) demonstrated that well-designed prompts can elicit improved reasoning from models, at times rivaling the benefits of fine-tuning. RAG methods (Lewis et al., 2020) help mitigate hallucinations by grounding responses in external documents, while instruction tuning (Mishra et al., 2022) enhances alignment with task-specific requirements.

Crucially, these approaches are not merely engineering hacks—they benefit from being grounded in an understanding of the mechanisms underlying LLM errors. Zhang et al. (2024) offer a compelling case study, identifying knowledge overshadowing as a key driver of what they term amalgamated hallucinations. This phenomenon occurs when a model trained on exclusively true statements still produces incorrect outputs by conflating multiple factual patterns. The root cause is an imbalanced training distribution, where high-frequency conditions suppress lower-frequency—but equally valid—ones.

Their analysis yields three core insights:

- Systematic error patterns: Hallucinations follow predictable generalization dynamics, reflecting the statistical dominance of certain patterns in the training data.
- Causal structure: These error patterns emerge from biased token prediction conditioned by asymmetric exposure during training.
- Corrective strategies: A decoding technique known as *self-contrastive decoding* can offset these effects at inference time, without additional model retraining.

Zhang et al.’s work exemplifies what the philosopher Bechtel (2008) calls *mechanistic explanation*: identifying components, mapping their interactions, and designing interventions to influence outcomes. Rather than relying on anthropomorphic labels like “hallucination,” their framework offers a clearer, system-level account of when and why certain failures emerge—and how they might be mitigated.

Still, these strategies have limits. As with boosting in human cognition, structural interventions do not eliminate foundational flaws; instead, they reshape inputs and task contexts to reduce error and enhance performance. That is precisely their strength. LLM task redesign, approached experimentally and informed by cognitive science, provides a principled way to test, probe, and refine model behavior. It enables us to study not just *what* models output, but how—and under what conditions—they succeed or fail.

## 6 LLM differences in thinking style

Another important lesson from research on human judgment comes from the study of individual differences. In particular, Stanovich’s (2011) work on rational thought highlights the variability in how people reason—emphasizing distinctions in cognitive style, thinking dispositions, and the capacity for reflective override. Much of this builds on the heuristics-and-biases tradition, yet Stanovich’s key contribution is to show that intelligence is not monolithic. He distinguishes between algorithmic-level intelligence (akin to IQ) and reflective-level rationality—the latter involving critical engagement with one’s beliefs and goals.

This distinction offers a compelling analogy for understanding differences among LLMs. Just as people vary in their susceptibility to biases or their willingness to engage effortfully with complex problems, different LLMs exhibit distinct “thinking styles” shaped by their architectures, training regimes, and fine-tuning methods. Some may excel at structured reasoning (e.g., OpenAI’s GPT-4), others shine in contextual interpretation (e.g., Claude 3.5), while still others trade raw capability for speed and deployability (e.g., Mistral L2 or DeepSeek-R1). Each model has its superpowers—and its blind spots.

This diversity matters for evaluation: a one-size-fits-all metric may fail to capture each model’s unique strengths and weaknesses. Recent work by Martin et al. (2021) demonstrates that these behavioral differences are often reflected in a model’s internal structure, revealing consistent patterns in how architectural and training choices shape model capabilities. Treating LLMs as interchangeable is as misleading as treating all human thinkers the same. Do different LLMs favor fluency over factuality? How do they respond under instruction pressure or in ambiguous contexts? Understanding and systematically comparing these tendencies - akin to studying cognitive styles in psychology - can help developers and users better match models to use cases and move toward a more principled science of evaluation.

## 7 Conclusion: toward an empirically grounded evaluation framework

Current LLM evaluation frameworks risk misalignment by over-relying on simplistic accuracy metrics and misleading metaphors. As argued throughout this paper, insights from human judgment research offer a pathway forward. Embracing lessons on heuristics, bounded rationality, and task design—while emphasizing mechanistic explanations, multi-dimensional accuracy models, and domain-sensitive evaluation strategies—can help build more robust evaluation frameworks for AI. By integrating such insights from cognitive science, AI assessment can evolve into a more rigorous, ecologically valid discipline, ensuring that future LLM development is driven by meaningful improvements rather than mere optimization for narrow benchmarks.

## Data Availability

The original contributions presented in the study are included in the article/supplementary material, further inquiries can be directed to the corresponding author.

## Footnotes

^1^While terms like “reasoning” and “hallucination” are widely used as convenient functional descriptors of model behavior, they should not be taken to imply that LLMs possess cognitive or experiential capacities akin to those of human minds. For a critique of such anthropomorphic metaphors—and of the conceptual risks involved in borrowing language between AI and brain sciences—see Floridi and Nobre (2024).
